# Observations on the Secretion of Saliva in a Case of Parotid Fistula

**Published:** 1880-11

**Authors:** 


### OBSERVATIONS ON THE SECRETION OF SALIVA IN A CASE OF PAROTID FISTULA.

A short communication from R. Harold A. Schofield, M. B., contains an account of a case which was placed at his disposal for purposes of experimentation.

In the course of these observations he found that the flow of saliva, produced by sugar, was more copious and lasted longer than that excited by salt, pepper, dry bread, water, milk, or lemonade. Salt, however, was found to be the most rapid in its action of all these substances, the flow of saliva beginning within five seconds after it had been taken into the mouth; dilute hydrochloric acid dilute acetic acid, oil of peppermint, oil of spearmint, and oil of cajeput, all excited a flow of saliva generally within from five to seven seconds after being dropped upon the tongue. Ether and chloroform by inhalation also produced salivation; that by the former being more copious and lasting longer than that caused by the latter. The excretion of iodide of potassium through the salivary glands was also proved to precede its elimination through the kidney. [Review of St. Bartholomew's Hospital Reports in Am. Jour. Med. Science.]

